# The Evolution of a Tick Bite Lesion

**DOI:** 10.7759/cureus.29865

**Published:** 2022-10-03

**Authors:** Jerome Goddard, Julie P Wyatt

**Affiliations:** 1 Biochemistry, Molecular Biology, Entomology, and Plant Pathology, Mississippi State University, Starkville, USA; 2 Dermatology, Wyatt Dermatology Clinic, Jackson, USA

**Keywords:** medically important parasites, tick bite, skin damage, parasitic infestations, lone star tick

## Abstract

Hard ticks (Acari: Ixodidae) use their mouthparts to cut through the epidermis and insert a barbed hypostome, leading to deep inflammation of local tissues. Herein, we describe cutaneous lesion development resulting from a tick bite at seven time points over a 30-day period. This case highlights the fact that ticks may produce lasting cutaneous lesions, which may persist for at least 30 days, even without any obvious pathology or complications.

## Introduction

Hard ticks (Ixodidae) use their mouthparts to cut through the epidermis and insert a barbed hypostome, which can lead to mixed, deep inflammation of local tissues. Tick bite lesions can occur anywhere on the body and may vary from pruritic papules to more chronic nodules [1]. Sometimes, a persistent nodule will develop at the bite site lasting six to 12 months and may histologically appear as a granuloma or lymphocytoma [2]. Another common tick bite reaction, especially seen in the Southern United States, is a raised annular rash very similar in appearance to the erythema migrans of Lyme disease. The CDC terms this "southern tick-associated rash illness (STARI)," which may be due to a hypersensitivity reaction caused by tick saliva or other unknown causes [3]. Skin manifestations of tick bites in humans have been described [1,4], although to our knowledge there has never been a careful description of an uncomplicated tick bite from the time of tick removal to the resolution of the lesion.

## Case presentation

**Figure 1:**
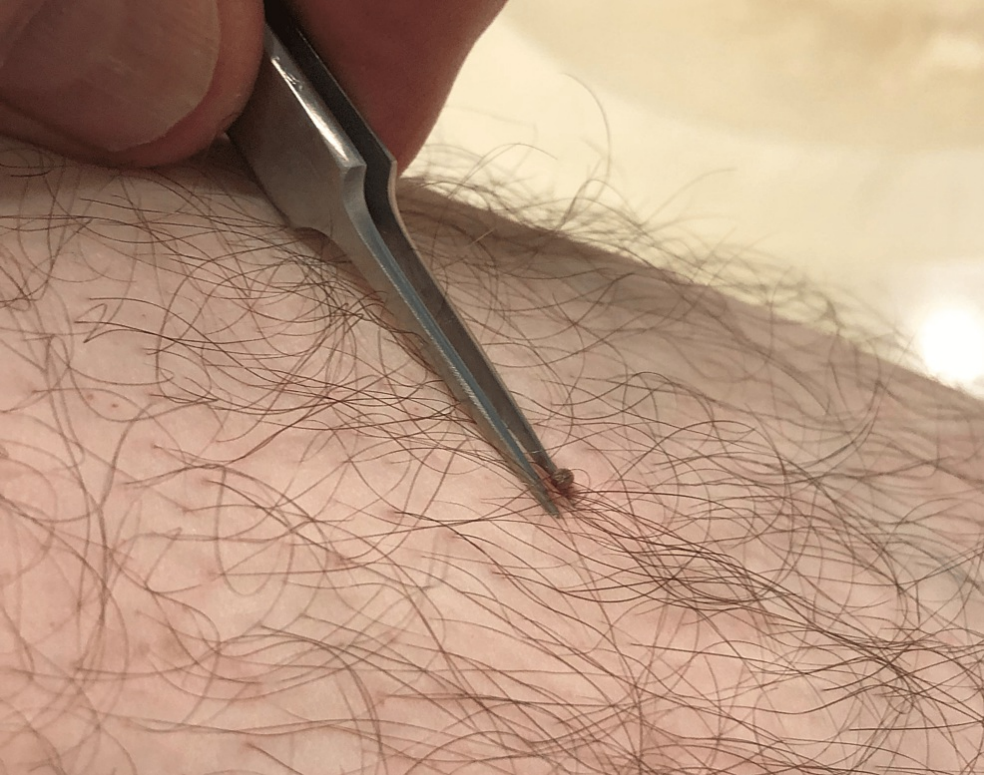
Nymphal-stage lone star tick (Amblyomma americanum) removed with a pair of fine-tipped forceps

**Figure 2:**
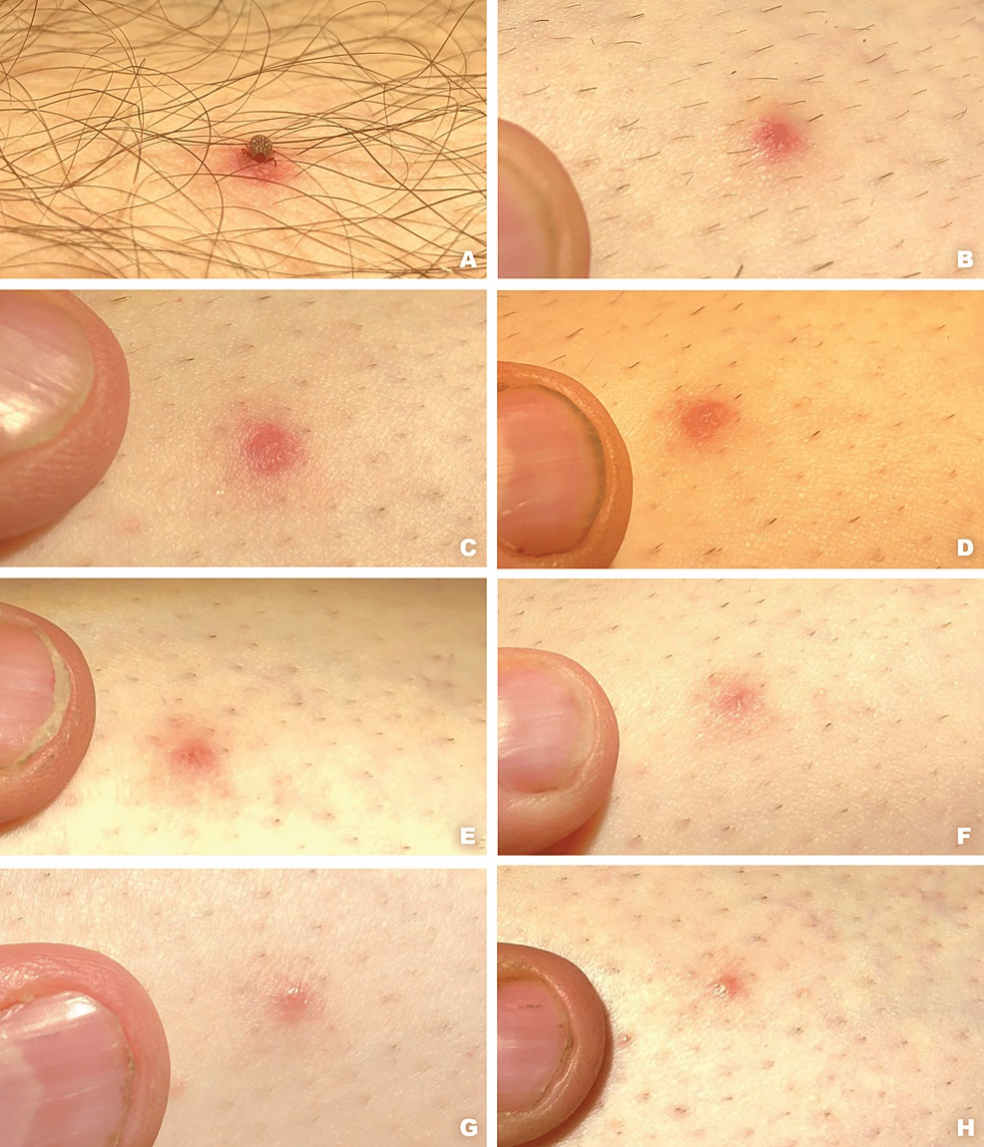
Photographs taken over the next 30 days Prior to removal, the tick is firmly attached by its mouthparts to the skin with a surrounding erythematous, evanescent macule (A); at 24 hours post removal, there is an erythematous, urticarial papule with faint concentric ecchymosis (B); at 48 hours, the erythematous, urticarial papule continues to be present (C); at 72 hours, the lesion is a persistent, erythematous papule (D); at 96 hours, the lesion has become an involuting erythematous macule with a central, pinpoint purpura (E); at 120 hours, there is pinpoint purpura with surrounding erythema (F); after 14 days, a light pink sclerotic papule remains (G); and at 30 days, only a small subtle pink papule remains (H)

Approximately one day after collecting ticks for a research project (June 2, 2022), the first author found an attached tick on his left leg about six inches below the knee. The tick was carefully photographed, removed with a pair of tweezers (Figure 1), and then taken to the lab for microscopic examination. Results revealed that it was a lone star tick (*Amblyomma americanum*) nymph. The bite site was lightly shaved to remove surrounding hair; then, photographs were made at seven additional time points over the next 30 days (Figure 2), during which the site was visually evaluated by the second author, a dermatologist. No treatments were applied; the lesion did not itch, nor was it ever warm to the touch (no signs of secondary infection). By 30 days, the lesion had healed uneventfully.

## Discussion

When ticks are firmly attached to a host, there is hemorrhage around the tip of the hypostome as a result of cytolytic and anticoagulant properties of their saliva [5]. The local lesion produced by ticks is initially a slightly red papule, developing into an inflammatory swelling with an erythematous halo (diameter of up to 4 cm) [5]. If undisturbed, ticks typically feed on blood for several days, causing them to swell to many times their usual size. In this study, the attached tick was removed from the patient after approximately one day, and no medical treatment was needed or applied. All subsequent lesion development was most likely due to saliva injected during the initial attachment process. For the first 48-72 hours after tick removal, there was a well-defined erythematous, urticarial papule that evolved to an involuting erythematous macule with a central, pinpoint purpura by 96 hours. At 120 hours post removal, there was pinpoint purpura with surrounding erythema. After 14 days, a light pink sclerotic papule remained, and at 30 days, only a small subtle pink papule remained.

## Conclusions

Tick bites cause vascular dilatation of dermal blood vessels. Lesion development is enhanced by host immune responses to tick salivary secretions. Lesions from tick bites have been reported to become pruritic and nodular, although that was not the case here. Annular lesions may indicate a condition termed STARI or perhaps the presence of Lyme disease. The case reported here highlights the fact that ticks may produce persistent cutaneous lesions, which may be present for at least 30 days, even without any obvious pathology or complications.
